# Towards an Accurate Estimation of COVID-19 Cases in Kazakhstan: Back-Casting and Capture–Recapture Approaches

**DOI:** 10.3390/medicina58020253

**Published:** 2022-02-08

**Authors:** Antonio Sarría-Santamera, Nurlan Abdukadyrov, Natalya Glushkova, David Russell Peck, Paolo Colet, Alua Yeskendir, Angel Asúnsolo, Miguel A. Ortega

**Affiliations:** 1 Department of Medicine, School of Medicine, Nazarbayev University, Nur-Sultan 020000, Kazakhstan; paolo.colet@nu.edu.kz (P.C.); alua.yeskendir@nu.edu.kz (A.Y.); 2 Departement of Mathematics, Statistics and Computer Sciences, University of Illinois at Chicago, Chicago, IL 60607, USA; nabduk2@uic.edu; 3 Department of Epidemiology, Biostatistics and Evidence-Based Medicine, Al-Farabi Kazakh National University, Almaty 050040, Kazakhstan; glushkovanatalyae@gmail.com; 4 Independent Researcher, 28410 Madrid, Spain; peckgarcia@gmail.com; 5 Department of Surgery, Medical and Social Sciences, Faculty of Medicine and Health Sciences, University of Alcalá, Alcalá de Henares, 28801 Madrid, Spain; angel.asunsolo@uah.es; 6 Ramón y Cajal Institute of Health Research (IRYCIS), 28034 Madrid, Spain; miguel.angel.ortega92@gmail.com; 7 Department of Medicine and Medical Specialties, Faculty of Medicine and Health Sciences, University of Alcalá, Alcalá de Henares, 28801 Madrid, Spain

**Keywords:** COVID-19, SARS-CoV-2, back-casting approach, capture–recapture method

## Abstract

*Background and Objectives:* Coronavirus disease 19 (COVID-19) has emerged as the most devastating syndemic of the 21st century, with worrisome and sustained consequences for the entire society. Despite the relative success of vaccination programs, the global threat of the novel coronavirus SARS-CoV-2 is still present and further efforts are needed for its containment and control. Essential for its control and containment is getting closer to understanding the actual extent of SARS-CoV-2 infections. *Material and Methods:* We present a model based on the mortality data of Kazakhstan for the estimation of the underlying epidemic dynamic—with both the lag time from infection to death and the infection fatality rate. For the estimation of the actual number of infected individuals in Kazakhstan, we used both back-casting and capture–recapture methods. *Results:* Our results suggest that despite the increased testing capabilities in Kazakhstan, official case reporting undercounts the number of infections by at least 60%. Even though our count of deaths may be either over or underestimated, our methodology could be a more accurate approach for the following: the estimation of the actual magnitude of the pandemic; aiding the identification of different epidemiological values; and reducing data bias. *Conclusions:* For optimal epidemiological surveillance and control efforts, our study may lead to an increased awareness of the effect of COVID-19 in this region and globally, and aid in the implementation of more effective screening and diagnostic measures.

## 1. Introduction

From its initial spread in China in December 2019, the coronavirus disease 2019 (COVID-19) has accounted for 173 million cases, and as of 8 June 2020 [1], more than 3.7 million confirmed deaths. Even with the rollout of effective vaccines, the severe acute respiratory syndrome coronavirus-2 (SARS-CoV-2) continues to threaten the world’s population—most of which remains susceptible to infection [2,3]. New emerging variants may not only be more transmissible but escape the immune response of previous infections and vaccination [4,5]. To monitor the dynamics of the pandemic and contain the spread of the virus, most countries have been publicly reporting daily counts of laboratory-confirmed cases and deaths, yet substantial undocumented infections have obscured the actual fraction of infected people. Although, also clearly identified from the beginning of the pandemic, were both the magnitude and relevance of asymptomatic cases [6]. The ultimate success of contact tracing for the containment of COVID-19 in the early stages of invasion relied on speed and efficacy and required that most secondary cases be discovered and isolated before they become infectious. The time from the primary case becoming infectious to the tracing of their contacts, therefore, needs to be less than the incubation period [7]. However, the large proportion of pre-symptomatic transmission, the limitations of testing capacity, in addition to the limitations of contact-tracing, continue to be critical weaknesses for the monitoring of the progression, and ultimately, for the control of the pandemic, because public health services are likely detecting only a small fraction of infections [8].

Both the unbiased estimation and the trends in disease incidence are necessary for epidemiological surveillance data and for the implementation of control efforts. A significant proportion of unreported cases is an important consideration. For COVID-19, these undocumented infections may have substantially contributed to virus transmission, explain the rapid spread of COVID-19, and its particularly challenging containment [9]; making it especially important to identify the gap between the actual number of people infected from the cases that do get reported. This gap was described during the early outbreak in China [9]. Although large-scale seroprevalence studies for the estimation of the actual number of infections found diagnosed cases to represent 10% of total cases [10], we still do not know how many individuals are currently infected. Understanding the extent of unreported infections is crucial the following: for situational awareness; for a reliable assessment of the specific stage of the epidemic and transmission (determining the progression of reported and unreported cases); and for how and when nonpharmaceutical interventions (NPIs) should be introduced, or relaxed [11].

The first two cases of COVID-19 in Kazakhstan were registered on 13 March 2020 [1]. Three days after the diagnosis of the first cases, an emergency regime was introduced for the period of 16 March 2020 to 15 April 2020, and later extended until 11 May 2020; but on 5 July 2020, the government had to introduce a 2nd lockdown [12]. Since then, four periods with diverse stringency of Non-Pharmaceutical Interventions (NPI) have been implemented. As of 14 June 2021, Kazakhstan has reported a total of 453,957 confirmed cases and 7465 deaths [1]. Our study presents a model with mortality data that are likely a more stable and reliable source of COVID-19 information, including the following: to estimate the underlying epidemic dynamic in Kazakhstan; account for the lag time from infection to death; and estimate the infection fatality rate.

## 2. Materials and Methods

Back-casting and Capture–recapture approaches were used to simulate and estimate the actual number of infected individuals in Kazakhstan.

### 2.1. Back-Casting Method

Back-casting is a statistical approach for the calculation of the actual cumulative number of infections of a population [13]. This method has been previously applied to estimate the actual extent of the infection in a series of countries [11,13]. With this approach, we assume that the mean time from infection to death follows a Gamma distribution with parameters α = (µ/s)^2^, β = (s^2^/µ) (µ denotes mean and s denotes standard deviation). It allows the use of the number of new daily deaths, together with a Gamma distribution, to make a backward projection in time of initial infection. We can calculate the estimated number of new infections that occurred on a day that has resulted in deaths on day *t* as follows:

(1) $$n_{i}\left(t^{\prime },t\right)=\frac{N_{f}\left(t\right)\;x\;f\left(t-t^{\prime };\;\alpha ,\beta \right)}{IFR}$$

*N_f_* (*t*): the number of new deaths to occur on day *t*.

*f* (*x*; *α*, *β*): the probability density function for the Gamma distribution.

*IFR*: infection fatality rate. The ratio of mortality to total infections, both symptomatic and asymptomatic [14].

Next, we can estimate the cumulative number of new infections on a given day by summing all as >. We then need to adjust it using the CDF of the Gamma distribution for the most recent day for which the number of deaths is known.

(2) $$N_{i}\left(t^{i}\right)=\frac{1}{F\left(t_{0}-t^{\prime };\;\alpha ,\;\beta \right)}\overset{t_{0}}{\underset{t=t^{\prime }+1}{\sum }}n_{i}\left(t^{\prime },t\right)$$

There is a limitation for the calculation of the total number of infected people on the last days of the period of time being analyzed, because some deaths may not have yet occurred: that means there might be an underestimation of deaths and of real infected people for those last days and the back-casting approach cannot fully project it to the number of infected people. Thus, to make a better prediction of the total number of infected people in those final days analyzed, it is better to use a back-casting approach up to some point and exponential smoothing (e.g., Holt linear) after that point to get a better prediction.

### 2.2. Capture–Recapture Method

The capture–recapture approach also uses the number of deaths to explore the total number of infected people. The primary difference from back-casting is that it permits the determination of only the estimation for a lower bound rather than the total number of affected individuals [15].

The implementation of this method requires using geometric distribution for the number of times a unit is identified in the sampling process. The Cauchy–Schwarz inequality formula is needed to estimate a lower bound. The formula to estimate unreported or hidden cases is the following:

(3) $$H_{t_{0}}=\overset{t_{m}}{\underset{t=t_{0}+1}{\sum }}\frac{\Delta N(t)[\Delta N(t)-1]}{1+\max(\Delta N(t-1)-\Delta D(t),0)}$$

∆*N* (*t*): number of new infections at day *t*.

∆*D* (*t*): number of new deaths at day *t.*

*t*0: starting day (day of the first reported COVID-19 case) tm: last day for which data are available. Using the above formula we can find a total size of infections: *N*($t_{m}$*) +*$H_{t_{0}}$

### 2.3. Data

The period of time covered by this work corresponds to March 2020 through April 2021. The cumulative number of tests performed per capita in the country was obtained from OurWorld in Data (https://ourworldindata.org/coronavirus). This database is updated daily, with the 5 May 2021 version used in this work.

## 3. Results

**Figure 1:**
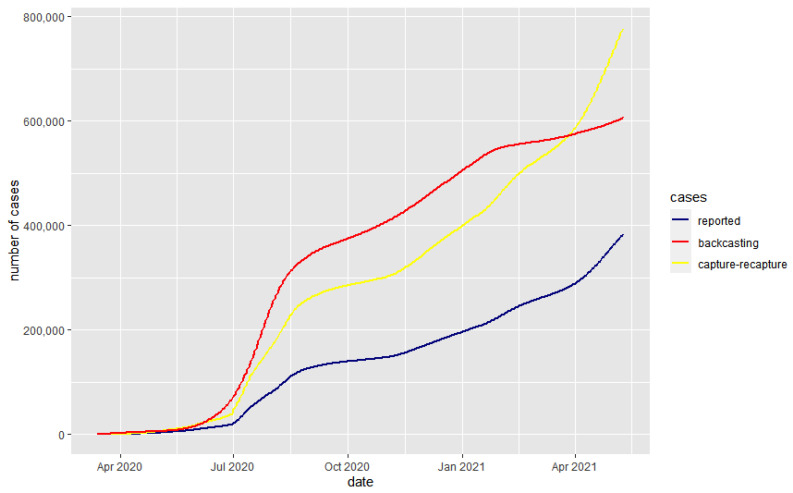
The cumulative number of COVID-19 cases.

**Figure 2:**
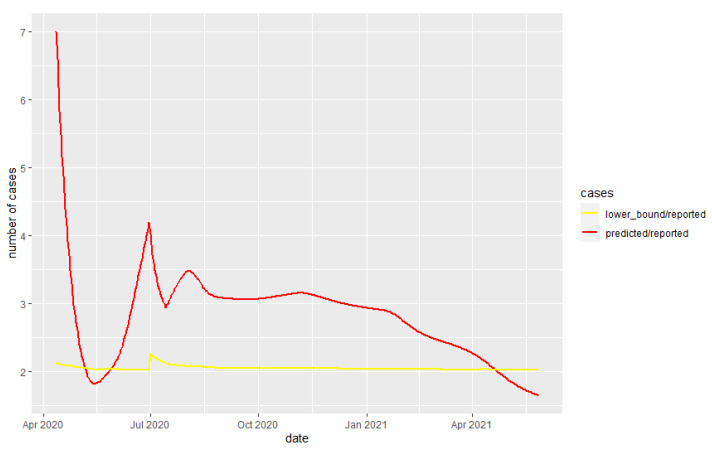
Ratio between number of cases for proposed methods and reported cases.

The back-casting and capture–recapture methods were applied for Kazakhstan. For the back-casting approach, *µ*, *s* and IFR must be estimated. Since IFR differs by age group [14], we used the method from Levin et al. to estimate an IFR for Kazakhstan of 0.005. Estimations for the *µ* and *s* are described by Philipp et al., 21.55 and 8.64, respectively [13]. Finally, using these methods, the result can be summarized with the plot below. As written earlier, we need to use exponential smoothing along with back-casting to obtain a more accurate result. In our case, *t* was 10 May 2021, and we used back-casting up to 21 March 2021 with exponential smoothing after that date. The gap of 50 days helps us to project almost all deaths to the number of total cases. Finally, using the above methods, the results are summarized in Table 1 and Figure 1 and Figure 2.

## 4. Discussion

The main finding of our work is the large proportion of undetected SARS-CoV-2 infections in Kazakhstan. These results are not surprising; a significant proportion of undocumented cases have also been estimated for other countries and with diverse methods [16].

Although established practices of infection control are particularly important to control the spread of SARS-CoV-2 transmission, such as isolation, contact tracing, and the use of personal protective equipment; a critical component of these efforts for the identification of asymptomatic but infectious cases is testing. For China, in the first months of the pandemic, probably less than 30% of cases were identified [17]. For the USA, likely more than 90% of infections have been undocumented [18,19]. Given the limitations of testing, several authors have proposed using deaths, which are assumed to be the least inaccurate of available measures of the extent of COVID-19 infections [11,20,21]. Here, we are updating the method proposed by Flaxman et al. [11] and Böhning et al. [15] which is based on the use of daily mortality data to develop a backward projection, in time, of an initial infection. The first approach used in our study, the back-casting method, was previously employed by Phipps et al. [13] to estimate the cumulative number of COVID-19 infections for 15 developed countries. The number of infections in those countries were found to be on average 6.2 times greater in their analysis, in comparison to the reported cases, although the differences were notably distinct between regions. By 31 August 2020 Belgium, France, Italy and the United Kingdom likely reported less than 10% of the actual number of cases. In comparison to this method, the capture–recapture approach is a lower bound estimator for the number of people affected by COVID-19.

Prior studies suggests that for various European countries on 18 April 2020 the estimated cases were on average 2.3 greater than those reported [15]. Simultaneously, Rochetti et al. [22] developed a model by which the capture recapture method could introduce an upper bound estimator for some European countries for similar dates, increasing the estimated cases by between 3.93 times for Norway, or of 7.94 times for France, thereby supporting the usefulness of these methods in the COVID-19 pandemic.

**Figure 3:**
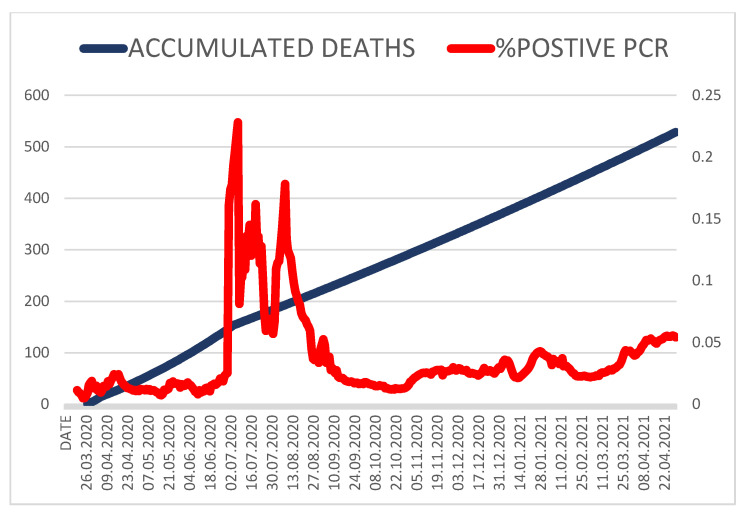
Test positivity percentage and accumulated number of reported deaths.

Our study suggests that regardless of the increased testing capabilities in Kazakhstan (Figure 3), official case reporting undercounts the number of infections by at least 60%. The main limitation of our estimation is that it relies heavily on the available estimation of the IFR, which is known to have large uncertainty. Figure 3 shows the dynamic percentage of positive PCR and of deaths reported during the pandemic. The pattern suggests that from July–December 2020 there may have been some anomalies in the process of reporting deaths. The accuracy of our estimation of actual cases would increase if the IFR estimate were optimized for a specific region and its uncertainty reduced. Depending on available datasets for each region, the estimation of actual cases can be improved by the inclusion of additional information such as daily positivity rates of diagnostic testing, or daily hospitalized cases. The increased awareness of COVID-19 among the general population has likely prompted people to seek medical care for respiratory symptoms, but a significant proportion of cases may remain asymptomatic [23]. A large increase in the identification and isolation of undocumented infections would be needed to fully control SARS-CoV-2. Over 50% of new SARS-CoV-2 infections were estimated to have originated from exposure to asymptomatic individuals [24]. Figure 3 also demonstrates the percentage of positive cases reported throughout the pandemic. The data indicate that during the first months of the pandemic, Kazakhstan experienced a critical deficit in testing. Yet later, the capacity for the identification of previously missed infections increased, probably reflecting an increased availability of testing.

In Kazakhstan, three days after the diagnosis of the first cases, an emergency decree was introduced from 16 March 2020 to 15 April 2020, including a ban on public and family events, closure of cinemas, theaters, shopping and entertainment centers, cleaning and disinfection of public spaces, and the establishment of restrictions on the entry and exit of various jurisdictions within the country. In-person classes were canceled at schools and universities and students were moved to online education. Due to the rapid increase in COVID-19 cases, the lockdowns, confinements, and stay-at-home orders were implemented in the two largest cities of Kazakhstan: Nur-Sultan and Almaty. Checkpoints were established at the points of entry to the cities. On 28 March, restrictive measures were extended within the cities to include the following: the closure of crowded places (parks, pedestrian streets etc.); the phased restriction of public transportation; the prohibition of meetings in public places by groups of more than three people; and movements of minors not accompanied by adults (Official Information Source of the Prime Minister of the Republic of Kazakhstan, 2020). From 1 April, Nur-Sultan and Almaty airports stopped accepting international and evacuation flights. The emergency decree was extended in the country until 11 May. After nearly two months, the restrictive measures began to be relaxed on 11 May 2020. As cases continued to increase after the relaxation, a new lockdown was introduced from 5 July through 17 August.

Although no serological studies have been conducted in Kazakhstan for the determination of prevalence, more than 50,000 cases of pneumonia were reported with COVID-19 like symptoms, but without a positive PCR [25]. Accurate surveillance of the COVID-19 pandemic is crucial for estimating key epidemiological values, such as the reproduction number, and hence the evaluation of the impact of control measures [26]. If reported case numbers do not reflect the shape and magnitude of the epidemic, it may bias the estimates of transmission potential and under or over-estimate the effectiveness of interventions.

The limitations of using deaths in our study may encompass various factors such as the number of deaths attributed to COVID-19 may be either over or undercounted. For example, for African countries, a marked under-estimation of COVID-19 deaths have been reported; yet other regions including the United States, the United Kingdom, and Spain have also likely under-reported the number of COVID-19 deaths in the first wave but over-estimated the mortality during the second and third wave, although these problems were starting to be addressed [27]. Of these issues, COVID-19 death underestimation is likely the most worrisome concern regarding the actual mortality data of the disease—with prior studies indicating that the number of deaths attributed to COVID-19 have been underestimated by at least 35% [28]. These underestimations may be due to differences in healthcare systems, case definitions, availability of testing, and other factors. The risk of suffering either a severe COVID-19 presentation or death, previous studies have found, is greater for Asian and black ethnicities [29]. For Kazakhstan, the ratio of severe to non-severe COVID-19 presentation, Yegorov et al. [30] reported, was about 5-fold lower in comparison to other countries, although these data only counted a third of all laboratory-confirmed COVID-19 cases in March to April 2020. The number of diagnosed cases and deaths attributable to COVID-19, is therefore likely to be more accurate for countries such as Kazakhstan than for other regions. Although there is still a global limitation for the estimation of the actual data of mortality caused by COVID-19, using these data are the most probable to be the least inaccurate method to unravel the real extent of SARS-CoV-2 infections over time.

The methods described in this work aim to provide an answer to a fundamental open question: “How many undetected cases are going around?”. The specific characteristic of SARS-CoV-2 that is transmissible 24–48 h before symptom onset and that in a significant proportion of cases remains asymptomatic generates significant limitations for public health authorities both to control the extension of the pandemic as well as to determine its real magnitude. As well as the specific limitations of both alternatives, a main limitation is common to both: they rely on the estimated IFR, which on many occasions is also complex to determine.

Both methods are easy to apply in practice, as they use time series of cumulative data, readily available from official data, and may provide a straightforward solution to shed light on undetected cases. Combining both methods allows to have a realistic estimation of the real magnitude of the infection. Back-casting and capture–recapture could be used to complement data obtained from testing as well as to inform the necessary forecasting modeling to understand the future dynamic of the pandemic.

## 5. Conclusions

We present statistical alternatives based on mortality data to describe the underlying epidemic dynamic in Kazakhstan and the most accurate number of infected individuals in Kazakhstan. These results may lead to an increased awareness of the impact of COVID-19 in this region and globally, aiding the implementation of screening and diagnostic measures for the achievement of more effective surveillance and control.

## Figures and Tables

**Table 1:** Number of cases for some dates.

Date (d/m/y)	Reported Cases	Capture–Recapture	Back-Casting
1 July 2020	20,743	47,005	83,491
1 October 2020	139,337	285,255	427,513
1 January 2021	196,471	400,647	576,962
1 February 2021	227,830	464,231	626,175
1 March 2021	258,254	524,604	638,869
1 April 2021	289,046	586,851	651,518
1 May 2021	359,887	729,996	674,266

## Data Availability

The data used to support the findings of the present study are available from the corresponding author upon request.
